# High-Glucose Microenvironment Accelerates Malignant Progression Via O-GlcNAcylation in Oral Squamous Cell Carcinoma

**DOI:** 10.1016/j.identj.2025.103897

**Published:** 2025-10-08

**Authors:** Zhuang Zhu, Wenhao Ren, Xiaohan Yan, Shaoming Li, Jingjing Zheng, Keqian Zhi, Ling Gao

**Affiliations:** a Department of Oral and Maxillofacial Reconstruction, The Affiliated Hospital of Qingdao University, Qingdao, China; b School of Stomatology, Qingdao University, Qingdao, China; c Key Lab of Oral Clinical Medicine, The Affiliated Hospital of Qingdao University, Qingdao, China; d Department of Oral and Maxillofacial Surgery, The Affiliated Hospital of Qingdao University, Qingdao, China; e Department of Endodontics, The Affiliated Hospital of Qingdao University, Qingdao, China

**Keywords:** Oral squamous cell carcinoma, Diabetes mellitus, High-glucose microenvironment, O-GlcNAcylation, PI3K/AKT/mTOR pathway

## Abstract

**Background:**

Diabetes mellitus (DM) is a risk factor for oral squamous cell carcinoma (OSCC). O-GlcNAcylation is a specific form of glycosylation modification sensitive to glucose levels and implicated in tumour progression. However, the association between the high-glucose environment, O-GlcNAcylation, and OSCC needs further exploration.

**Methods:**

The relationship between DM status and clinicopathological factors was analysed. The Kaplan–Meier analysis was performed to evaluate the effect of DM on OSCC prognosis. The proliferation, migration, invasion, and apoptosis of OSCC cell lines (SCC25 and CAL27) in the high-glucose microenvironment were analysed using CCK8, colony formation, wound healing, transwell assays, flow cytometry, and western blots (WB). Immunohistochemical staining and WB were used to detect O-GlcNAc transferase (OGT) and O-GlcNAcylation levels in OSCC tissues and cells. Changes in cell proliferation, migration, invasion, and apoptosis were analysed in OSCC cells after OGT-shRNA knockdown or adding the OGT inhibitor OSMI-1. The effect of O-GlcNAcylation on the expression of phosphorylated proteins of the PI3K/AKT/mammalian target of rapamycin signalling pathway was analysed by WB.

**Results:**

DM status was associated with the clinical T stage, lymph node metastasis, Ki-67, and depth of invasion in OSCC. DM status was significantly associated with prognosis in patients with OSCC. In vitro experiments showed that the high-glucose microenvironment promoted the malignant progression of OSCC cells. OSCC tissues from patients with DM and OSCC cells cultured in high-glucose medium exhibited high O-GlcNAcylation levels. Decreases in hyper-O-GlcNAcylation by knocking down or inhibiting OGT inhibited proliferation and metastasis and promoted the apoptosis of OSCC cells in a high-glucose environment. The hyper-O-GlcNAcylation-mediated protumoural properties partially depended on the PI3K/AKT/mammalian target of rapamycin pathway.

**Conclusions:**

The findings highlight the elevated expression of O-GlcNAcylation in OSCC in a high-glucose microenvironment and its involvement in tumour malignant progression. The study findings have significant implications for the treatment of patients with OSCC who also have DM.

## Graphical abstract

**Figure:**
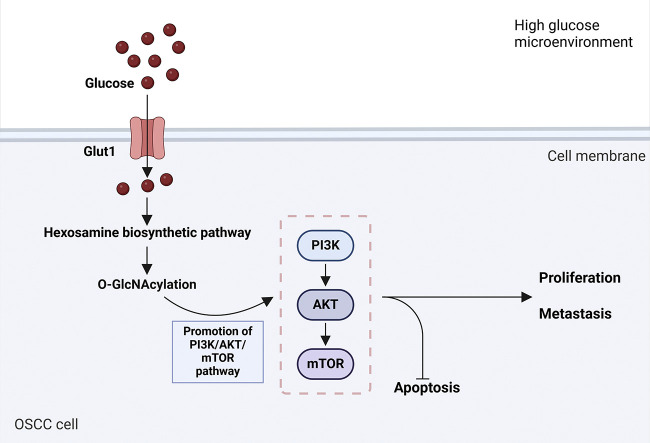

## Background

Oral squamous cell carcinoma (OSCC) is the most common and severely manifested malignant tumour in the oral and maxillofacial regions.^1^ In 2020 alone, there were an estimated 170,000 deaths and over 370,000 new cases of oral cancer worldwide.^2^ Several risk factors, such as smoking, excessive alcohol consumption, betel quid chewing, obesity, and diabetes mellitus (DM), are currently believed to be closely associated with OSCC tumourigenesis and progression.^3^, ^4^, ^5^, ^6^ Although DM is recognized as a risk factor for OSCC, its impact on patient prognosis and related mechanisms remains unclear.

Hyperglycaemia is the main feature of DM, and the clinical control of hyperglycaemia can prevent the development of diabetic complications.^7^, ^8^, ^9^ Studies have shown that cancer cells exposed to high levels of glucose develop altered intracellular signalling and malignant cancer phenotypes.^10^^,^^11^ A high-glucose microenvironment promotes the progression of several cancer cell types through various mechanisms and pathways dependent on aerobic glycolysis, such as the hexosamine biosynthetic pathway (HBP), are upregulated in cancer cells under high glucose conditions.^12^^,^^13^ The HBP converts glucose to UDP-*N*-acetylglucosamine (UDP-GlcNAc), a substrate for O-GlcNAcylation. O-GlcNAc transferase (OGT) plays a pivotal role in O-GlcNAcylation; it adds an O-GlcNAc moiety to the free hydroxyl group of specified serine and threonine residues on proteins. This process is reversed by O-GlcNAcase. O-GlcNAcylation modifies many intracellular proteins, linking nutrient fluxes to cellular processes and playing a crucial role in maintaining important cellular processes such as cell growth, survival, and invasion.^14^, ^15^, ^16^

In this study, we hypothesized that a high-glucose microenvironment promotes the proliferation and metastasis of OSCC while inhibiting apoptosis, primarily through the regulation of O-GlcNAcylation and OGT, thereby accelerating tumour progression. To investigate this hypothesis, we first performed a retrospective clinical analysis to assess the impact of DM on the prognosis of OSCC patients, with the aim of determining whether hyperglycaemia-related metabolic conditions are associated with poorer clinical outcomes. These clinical findings provided a rationale for exploring the direct effects of a high-glucose microenvironment on OSCC cells. We then conducted a series of in vitro experiments using OSCC cell lines cultured under high-glucose conditions to examine how elevated glucose levels influence key malignant behaviours of OSCC cells and to evaluate the potential role of O-GlcNAcylation in mediating these effects. Overall, this study aimed to elucidate the interplay between a high-glucose microenvironment, O-GlcNAcylation, and OSCC progression, shedding light on the mechanisms linking DM with OSCC and offering potential therapeutic insights for OSCC patients with comorbid DM.

## Materials and methods

### Clinical data collection

We selected 394 consecutive patients with primary OSCC who underwent the extensive resection of primary cancer at the Department of Oral and Maxillofacial Surgery, the Affiliated Hospital of Qingdao University, between July 2017 and February 2023. The inclusion criteria were^1^ primary OSCC,^2^ no previous tumour immunotherapy, radiotherapy, or chemotherapy, and^3^ complete clinicopathological and follow-up data. The exclusion criteria were^1^ OSCC with distant metastasis,^2^ poor staining quality or insufficient tumour tissue, and^3^ patients lost to follow-up. The research adhered to the Declaration of Helsinki’s standards and received approval from the Ethics Committee of the Affiliated Hospital of Qingdao University (QYFYWZLL 30079).

### Immunohistochemistry

Paraffin-embedded tissues were sectioned at a thickness of 4 µm and utilized for immunohistochemical (IHC) analysis. Antigen retrieval was performed at 95°C for 20 minutes with 10 mM sodium citrate (pH 6.0). Subsequently, endogenous peroxidase was blocked for 10 minutes with 3% hydrogen peroxide and goat serum and incubated with primary antibodies overnight at 4°C. DAB (Sangon Biotech) was employed to observe tissue antigens. Two independent investigators visualized specific antigens using a light microscope (Olympus) in 10 randomly selected fields. The staining intensity was evaluated using the *H*-score method: *H*-score = ∑ (*P_i_* × *i*), where *P_i_* is the percentage of cells with staining intensity and *i* is the staining intensity score (0 = negative, 1 = weak, 2 = moderate, 3 = strong).

### Cell culture and infection

Two human OSCC cell lines (SCC25 and CAL27) were procured from Procell. The cell lines were cultured in Dulbecco’s Modified Eagle’s Medium containing 5.5 or 25 mM d-glucose, supplemented with 10% foetal bovine serum and 1% penicillin-streptomycin (Procell). The cell lines were maintained in a cell culture incubator at 37°C with 95% humidified air and 5% CO₂. For infection, OGT shRNA knockdown lentiviral particles and their controls were obtained from Genechem. OSCC cell lines were infected with lentiviral particles and then selected with puromycin. Western blots (WB) confirmed the stable knockdown of OGT.

### CCK-8

The treated OSCC cells were added to 96-well plates (1 × 10^3^ cells/well). The cells were incubated in a medium containing different glucose concentrations, and 10 μL of CCK-8 (Solarbio) was added at 0, 24, 48, and 72 hours. After 2 hours of incubation, the optical density values were monitored using a Multiskan FC Microplate Reader (Thermo Fisher Scientific, Inc.) at 450 nm.

### Colony formation assay

The treated OSCC cells (500 cells) were inoculated into 6-well plates and incubated in a medium containing different glucose concentrations for 14 days. A 3% paraformaldehyde solution (Elabscience) was used to fix and stain the attached clones after staining with 0.1% crystal violet (Solarbio), and the clones were counted.

### Wound healing assay

Cells were inoculated into six-well culture dishes and cultured at 37°C and 5% CO_2_. When the cell monolayer reached 95% to 100% confluence, the medium was removed from the dishes, and they were rinsed twice with phosphate-buffered saline. The cell monolayer was scratched in a straight line through the centre of the dish using a sterile P-200 pipette tip. The exact position and focal plane were determined, and images were acquired at 0, 12, and 24 hours.

### Transwell assay

Transwell inserts (Biofil) with a filtration accuracy of 8 μm were used for the Transwell assay. The wells were left uncoated or coated with 50 μL of Matrigel on the lower surface of the Transwell membrane and incubated at 37°C for 30 minutes to gelatinize. A total of 5 × 10^4^ cells were seeded in the upper chamber with or without Matrigel, and 500 μL of specified complete medium was added to the lower chamber. After 12 hours of incubation, the cells were fixed with 4% paraformaldehyde and stained with 0.1% crystal violet.

### Flow cytometry

After 48 hours of incubation, the cells were collected and prepared as a cell suspension with trypsin. Then, the cell suspension was transferred to a centrifuge tube, rinsed with phosphate-buffered saline, and moved to an Eppendorf tube. The cells were stained for 20 minutes using the Annexin V-FITC/PI Apoptosis Detection Kit (Elabscience). Finally, the percentage of apoptotic cells was detected by flow cytometry.

### Western blots

The cells were lysed with radioimmunoprecipitation assay buffer. Bicinchoninic acid kits (Elabscience) were used to determine total protein concentrations. Total protein was separated by sodium dodecyl sulphate-polyacrylamide gel electrophoresis (Epizyme) at 80 V for 30 minutes and then 120 V for 60 minutes. Then, the proteins were transferred to a polyvinylidene fluoride membrane (Merck Millipore) at 290 mA for 90 minutes. Skim milk (5%) was applied for blocking. The membranes were incubated overnight at 4°C with specific primary antibodies. The membranes were washed with Tris-buffered saline with Tween-20 and incubated with horseradish peroxidase (HRP)-linked secondary antibodies. Finally, all protein bands were measured by adding an enhanced chemiluminescence reagent (Yeasen). The experiments were repeated three times independently. The representative blots from three independent experiments were provided in the figure.

### Antibodies and reagents

Antibodies against N-cadherin (N-cad) (1:1000 for WB, HY-P80238), E-cadherin (E-cad) (1:1000 for WB, P80113), vimentin (1:1000 for WB, HY-P80371), Bcl2 (1:1000 for WB, P80566), Bax (1:1000 for WB, P80028), PI3K (1:1000 for WB, HY-P80867), and phosphorylated P13K (p-PI3K) (1:1000 for WB, HY-P80846) were purchased from MedChemExpress; antibodies against AKT (1:1000 for WB, 4691T) and p-AKT (1:1000 for WB, 13038T) were purchased from Cell Signaling Technology; antibodies against OGT (1:5000 for WB, 1:250 for IHC, 66823-1-Ig), mammalian target of rapamycin (mTOR) (1:5000, 66888-1-Ig), p-mTOR (1:5000, 67778-1-Ig), and β-Actin (1:20,000 for WB, 66009-1-Ig) were obtained from Proteintech; and the O-GlcNAc antibody (1:1000 for WB, 1:200 for IHC, MA1-072) was purchased from Invitrogen. The secondary antibodies, HRP-linked antirabbit IgG (1:5000, E-AB-1003) and HRP-linked antimouse IgG (1:5000, E-AB-1001), were purchased from Elabscience. OSMI-1 (HY-119738) was purchased from MedChemExpress.

### Statistical analysis

Statistical analyses were performed using SPSS 29.0 (IBM Corporation) and GraphPad Prism (Version 9.5). The Chi-squared test was used to examine the link between DM status and clinicopathological elements. The Kaplan–Meier survival curve and log-rank test were used to assess patient survival outcomes. The results were expressed as the mean ± standard deviation. Statistical differences were assessed using the Student’s *t* test (two‐tailed). Multi‐group analyses of more than two groups were performed using ANOVA.

## Results

### Relationship between DM and the clinicopathologic characteristics of patients with OSCC

**Fig. 1:**
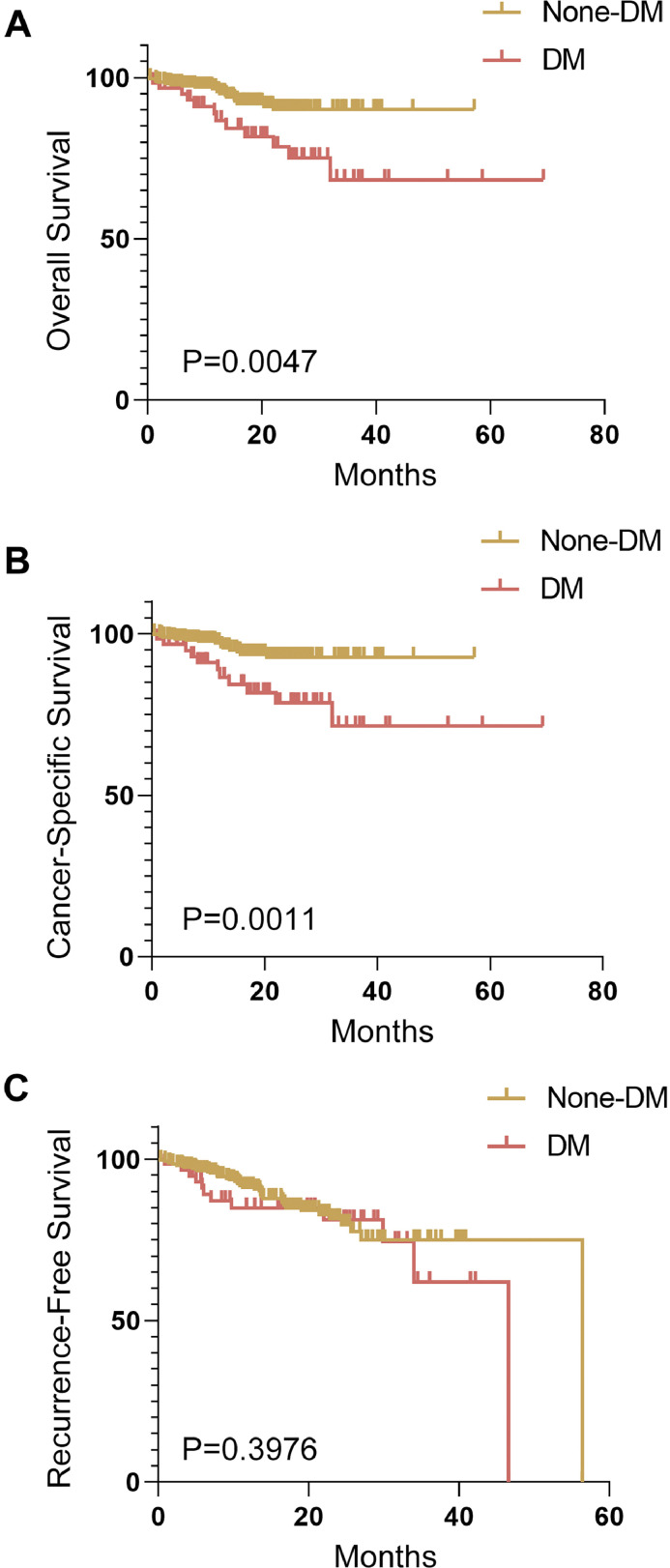
Prognosis of patients with OSCC according to DM status. (A) Overall survival. (B) Recurrence-free cancer-specific survival. (C) Recurrence-free survival.

A total of 394 consecutive patients were included in the study and divided into two groups according to DM status. The relationship between DM status and clinicopathological characteristics was then analysed. As shown in Table, a diagnosis of DM was positively associated with age, clinical T stage, lymph node metastasis, Ki-67 (a marker of tumour proliferation and local invasion), and the depth of invasion, but not with gender, smoking, drinking, or differentiation. The Kaplan–Meier method and log-rank test showed that patients with DM had poorer overall survival and cancer-specific survival than those without DM (Figure 1A,B). However, no significant difference in recurrence-free survival was found between the two groups (Figure 1C). Together, these results suggest that DM status is strongly associated with poor prognosis in patients with OSCC.

**Table:** Relationship between diabetes mellitus (DM) status and clinicopathological features.

Characteristic	None-DM (*n* = 332)	DM (*n* = 62)	*χ*^2^/*z*	*P*
**Age (y)**				
<60	141 (88.68)	18 (11.32)	3.919	***.048***
≥60	191 (81.28)	44 (18.72)		
**Gender**				
Male	239 (85.36)	41 (14.64)	0.872	.350
Female	93 (81.58)	21 (18.42)		
**Smoking**				
Yes	163 (87.17)	24 (12.83)	2.260	.133
No	169 (81.64)	38 (18.36)		
**Drinking**				
Yes	133 (84.18)	25 (15.82)	0.001	.969
No	199 (84.32)	37 (15.68)		
**Clinical T stage**				
T1-T2	202 (87.45)	29 (12.55)	4.263	***.039***
T3-T4	130 (79.75)	33 (20.25)		
**Lymph node metastasis**				
N–	214 (88.07)	29 (11.93)	6.912	***.009***
N+	118 (78.15)	33 (21.85)		
**Ki-67**				
<40%	177 (88.94)	22 (11.06)	6.644	***.010***
≥40%	155 (79.49)	40 (20.51)		
**Differentiation**				
Poorly	33 (84.62)	6 (15.38)	–0.608	.543
Moderately	154 (85.56)	26 (14.44)		
Well	145 (82.86)	30 (17.14)		
**Depth of invasion**				
≤10	208 (87.39)	30 (12.61)	4.444	***.035***
>10	124 (79.49)	32 (20.51)		

Significant results are highlighted in bold and italics, with a significance level of *P* < .05.

### High glucose promotes proliferation and metastasis and suppresses apoptosis in OSCC cells

**Fig. 2:**
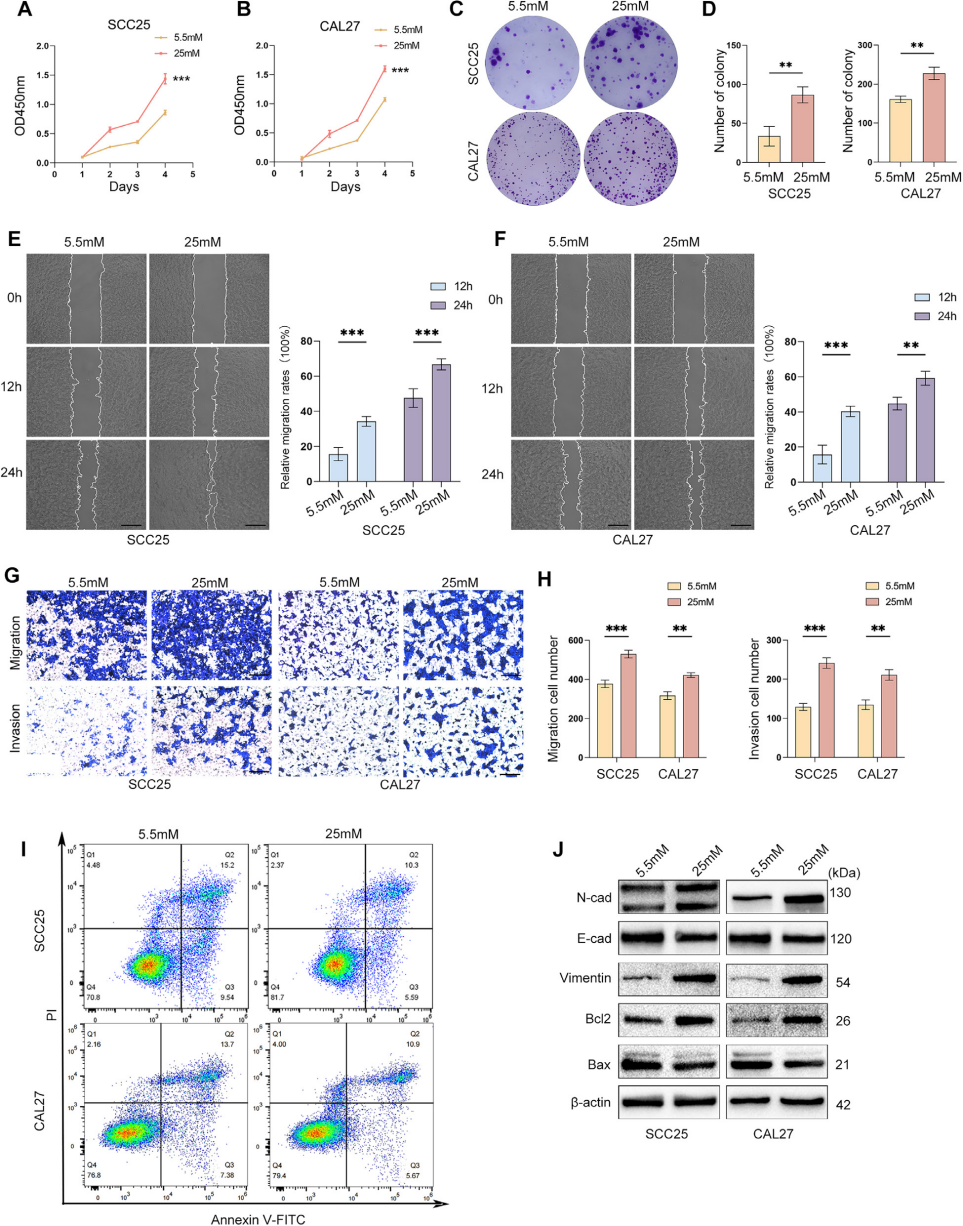
High glucose promotes proliferation and metastasis and suppresses apoptosis in OSCC cells. (A and B) SCC25 and CAL27 cells were cultured in 5.5 and 25 mM glucose for 48 hours. At the indicated time points (24, 48, and 72 hours), cell viability was measured using the CCK-8 and (C and D) colony formation assays. The migration and invasion abilities of OSCC cells were assessed by (E and F) wound healing and (G and H) Transwell assays after treatment with different glucose concentrations. Scale bars: 200 μm. (I) OSCC cell apoptosis was measured by flow cytometry following exposure to a medium containing different glucose concentrations. (J) OSCC cells were treated with different concentrations of glucose for 48 hours, and Western blots were performed to detect the expression of N-cadherin, E-cadherin, vimentin, Bax, and Bcl-2. β-actin was used as the internal loading control. *n* = three independent experiments. Data were presented as the mean ± SD. *P* values were determined by two-way ANOVA (A and B) or the two-sided *t* test (D, E, F, H). *P < .05, **P < .01, ***P < .001, ****P < .0001.

Given the important role of DM status in the prognosis of OSCC patients, we further explored the biological effects of a high-glucose microenvironment on OSCC cells through in vitro experiments. SCC25 and CAL27 cells were cultured in a medium with different concentrations of glucose to mimic standard and high-glucose microenvironments. CCK-8 assays were used to examine the relationship between high glucose and the proliferation of OSCC cells. The results indicated that high glucose potently promoted the growth of OSCC cells (Figure 2A,B). Regarding colony formation, the number of SCC25 and CAL27 colonies was significantly increased in high-glucose medium compared to the standard glucose counterpart. Representative images are provided in Figure 2C,D. Subsequently, we examined the migratory and invasive abilities of SCC25 and CAL27 cells using a wound healing assay. The results revealed that the migratory ability of OSCC cells in the high-glucose group was significantly increased compared with the standard glucose group (Figure 2E,F). In addition, the Transwell assay exhibited a consistent tendency towards increased OSCC cell migration and invasion in high-glucose medium (Figure 2G,H). Epithelial-mesenchymal transition (EMT) enables epithelial cells to acquire migratory and invasive capabilities.^17^ Therefore, we further examined the effects of glucose supply on EMT- and metastasis-associated markers using WB and found that N-cad and vimentin were increased in the high-glucose group, and E-cad was decreased (Figure 2I-K). Flow cytometry was used to detect the effects of the high-glucose microenvironment on OSCC cell apoptosis. The results showed that the apoptosis of SCC25 and CAL27 cells was suppressed in high-glucose medium (Figure 2L, Supplementary Figure S1C). We also measured the expression level of apoptosis indexes. High glucose promoted the expression of Bcl-2 and suppressed that of Bax compared to standard glucose (Figure 2J, Supplementary Figure S1A,B). Our results suggest that high glucose promotes proliferation and metastasis and suppresses apoptosis in OSCC cells.

### High glucose enhances O-GlcNAcylation and the expression of OGT in OSCC specimens and cell lines

**Fig. 3:**
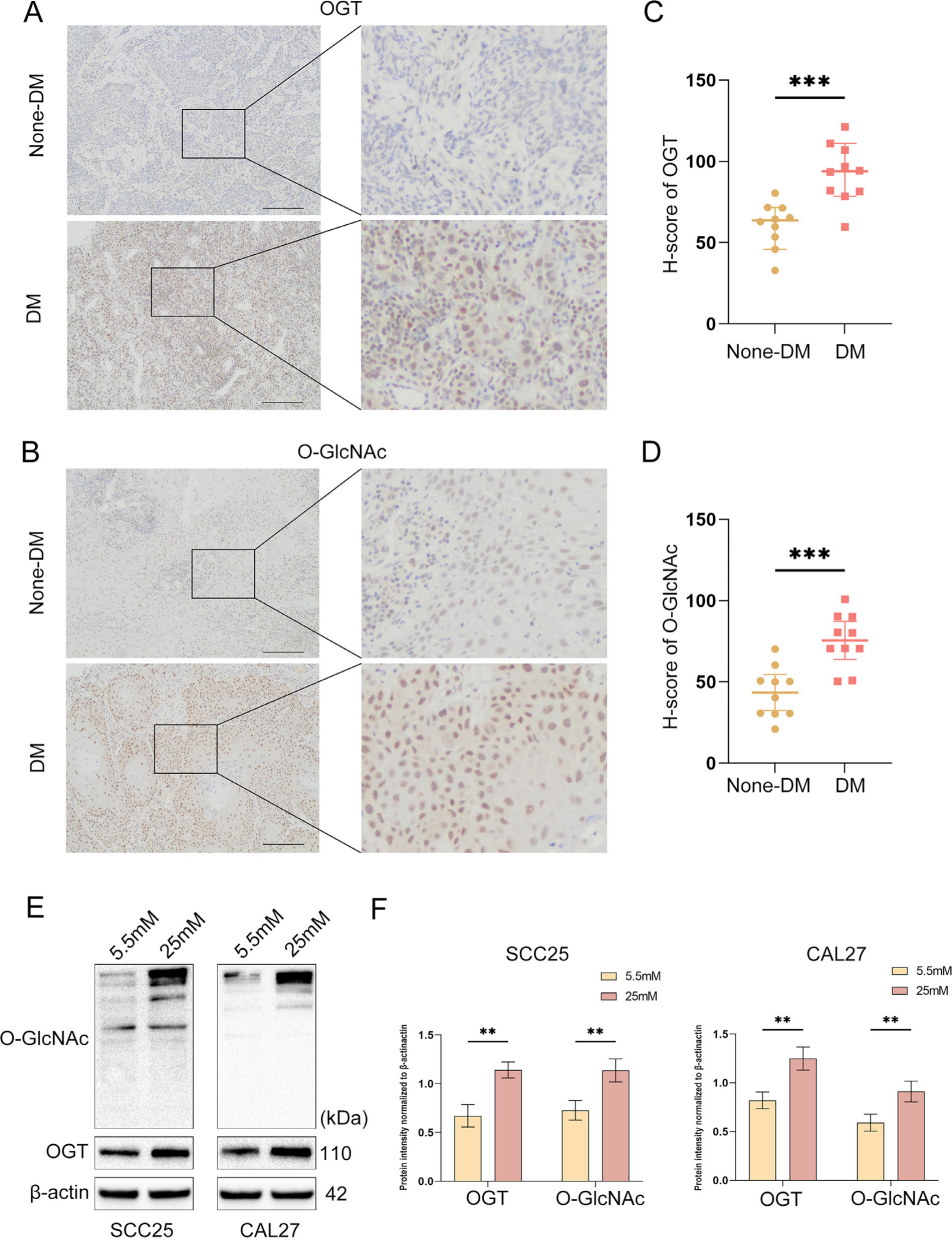
O-GlcNAcylation and OGT expression were upregulated in OSCC cell lines and tissue specimens in a high-glucose microenvironment. (A and B) O-GlcNAcylation and OGT expression in the OSCC tissues of patients with and without DM. Scale bars: 200 μm. (C and D) Statistical analysis of the two groups (*n* = 10 for each group). (E and F) The expression of O-GlcNAcylation and OGT in OSCC cells exposed to a medium containing 5.5 and 25 mM glucose was determined by Western blots. *n* = three independent experiments. Data were presented as the mean ± SD. *P* values were determined by the two-sided *t* test. *P < .05, **P < .01, ***P < .001.

Although the findings indicate that glucose supply is crucial for proliferation, metastasis, and apoptosis in OSCC, less is known about the underlying molecular mechanisms in this context. O-GlcNAcylation is a nutritional sensor that is sensitive to nutrient availability in the tumour microenvironment. First, we investigated the expression levels of O-GlcNAc and OGT in OSCC tissues from patients with and without DM using IHC to clarify whether O-GlcNAcylation participates in the protumoural effects of the high-glucose microenvironment in OSCC. The levels of O-GlcNAc and OGT were significantly higher in the tissues of patients with DM than in those without DM (Figure 3A-D). Next, WB were performed to examine O-GlcNAcylation and OGT levels in OSCC cells. SCC25 and CAL27 OSCC cell lines treated with high glucose showed much higher O-GlcNAcylation and OGT levels than when cultured in a medium with standard glucose (Figure 3E,F). These data suggest that O-GlcNAcylation and OGT expression in OSCC are positively regulated by glucose concentrations, which might underlie the protumoural activities of DM in OSCC.

### Decreases in O-GlcNAcylation inhibit OSCC cell proliferation and metastasis and promote cell apoptosis

**Fig. 4:**
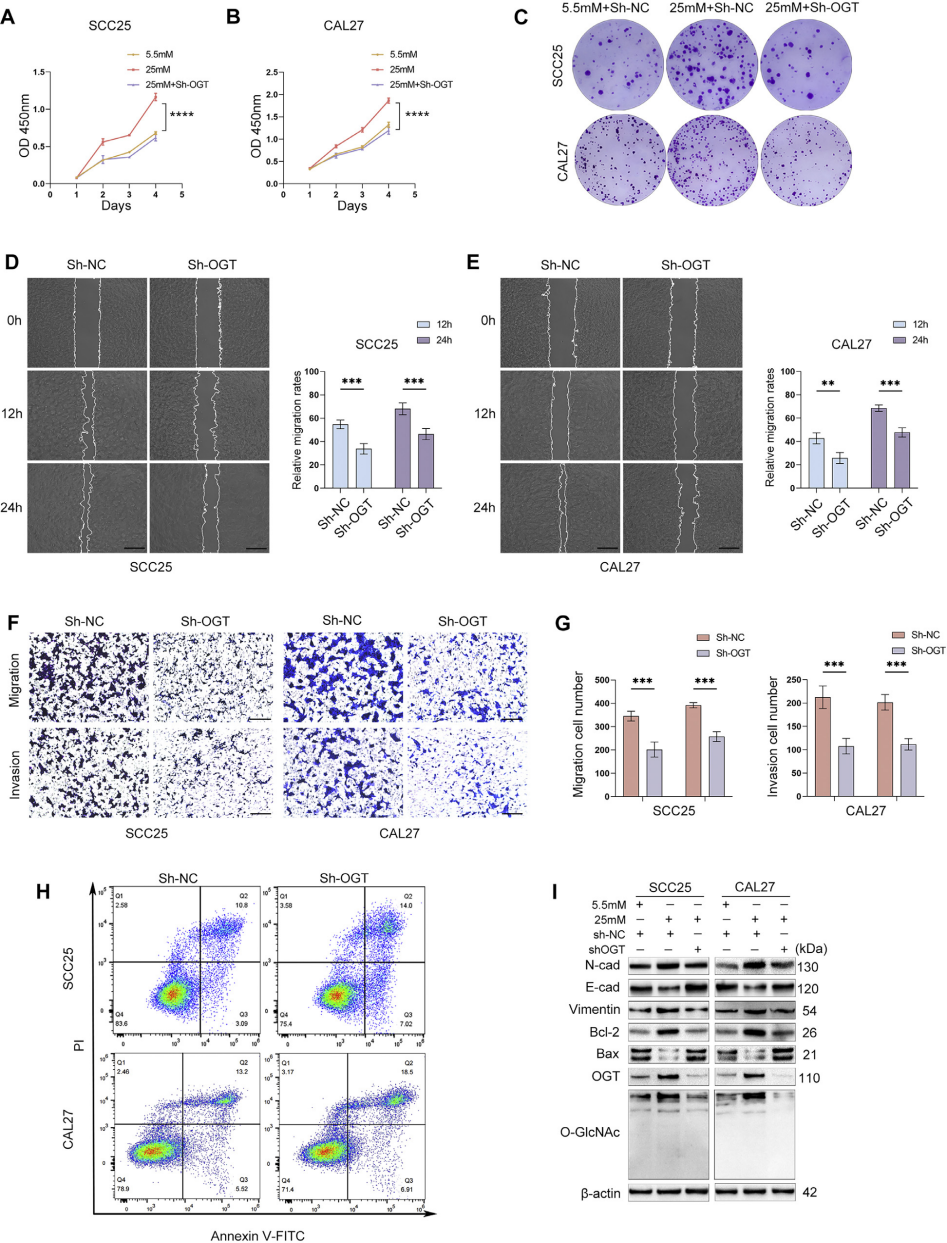
OGT downregulation inhibits proliferation and metastasis and enhances apoptosis in OSCC cells. The proliferation of SCC25 and CAL27 cells treated with 25 mM glucose and/or OGT knockdown was determined using CCK-8 (A and B) and colony formation (C) assays. (D and E) Wound healing and (F and G) Transwell assays were used to detect the migratory and invasive abilities of OSCC cells after OGT knockdown at 25 mM glucose. Scale bars: 200 μm. (H) SCC25 and CAL27 cell apoptosis was measured by flow cytometry following OGT knockdown in 25 mM (high) glucose. Expression of O-GlcNAcylation, OGT, and representative EMT and cell apoptosis markers of OSCC cells exposed to a medium containing 25 mM glucose and/or OGT knockdown were examined using (I) Western blot analysis. *n* = three independent experiments. Data were presented as the mean ± SD. *P* values were determined by two-way ANOVA (A and B) or the two-sided *t* test (D, E, G). *P < .05, **P < .01, ***P < .001.

We further investigated whether the protumoural properties of high glucose in OSCC exert their influence through O-GlcNAcylation regulation. For this purpose, we established stable SCC25 and CAL27 cell lines with OGT knockdown. The CCK‑8 and colony formation assay results revealed that the significant increase in cell proliferation stimulated by high-glucose treatment was prevented by OGT knockdown (Figure 4A-C, Supplementary Figure S1D). Consistently, the inhibition of OGT activity by the small molecule inhibitor OSMI-1 prevented cell proliferation (Supplementary Figure S2A,B). The migratory and invasive capabilities were suppressed by OGT knockdown or inhibition in high-glucose conditions (Figure 4D-G, Supplementary Figure S2C-F). Flow cytometric analysis showed that OGT knockdown prevented the inhibitory effect of high glucose on apoptosis (Figure 4H, Supplementary Figure S1G). As expected, the Western blotting assays confirmed that potent OGT inhibition reduced the expression level of N-cad, vimentin, and Bcl-2 and increased that of E-cad and Bax (Figure 4I, Supplementary Figures S1E,F and S2G-I), indicating that OGT knockdown had a reversible effect on proliferation and metastasis promotion and apoptosis inhibition induced by high glucose.

### The effect of high glucose on cell proliferation, metastasis, and apoptosis partially depends on the PI3K/AKT/mTOR signalling pathway

**Fig. 5:**
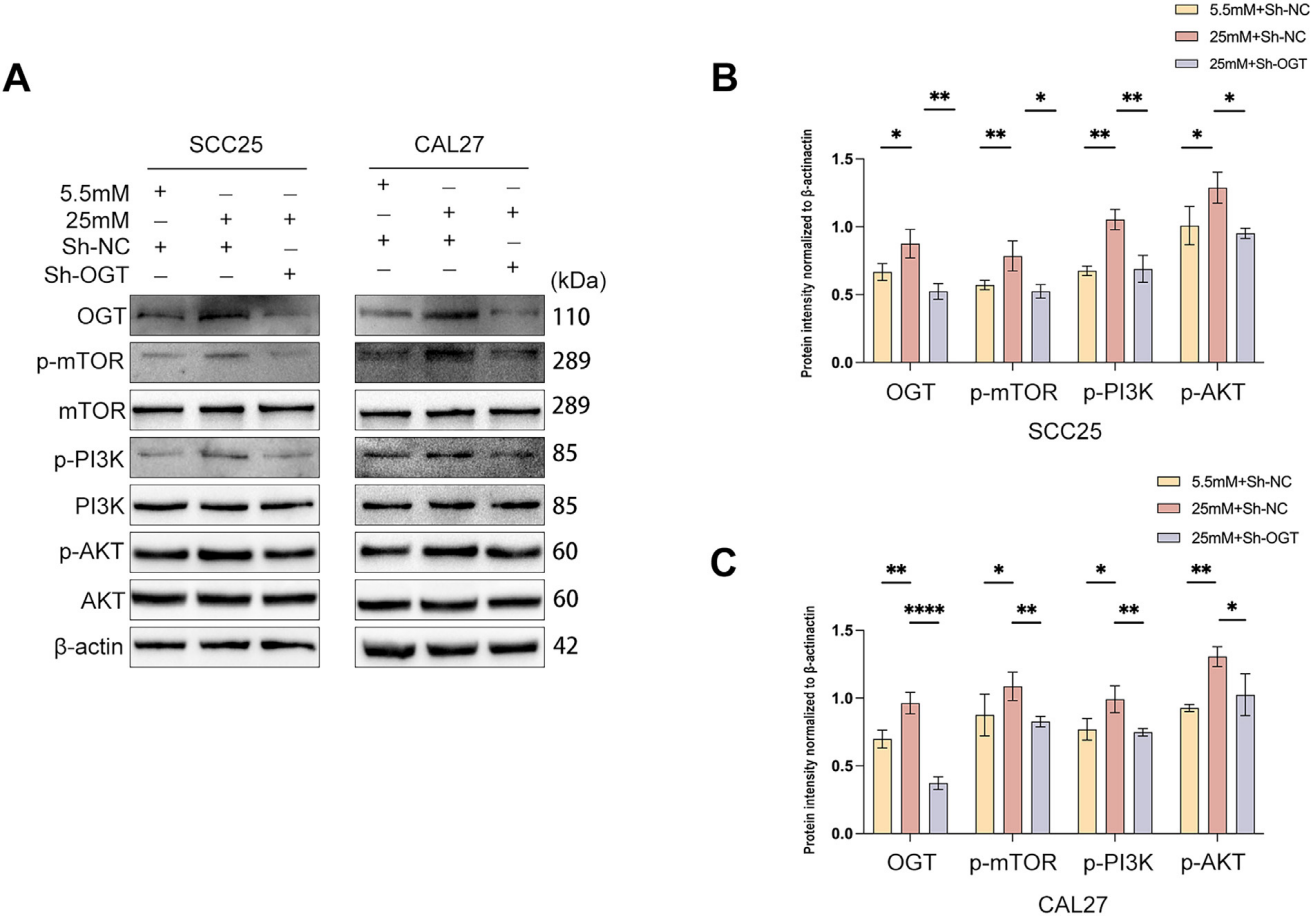
The effects of OGT on cell proliferation, metastasis, and apoptosis partially depend upon the PI3K/AKT/mTOR signalling pathway in OSCC cells. (A-C) Levels of PI3K, AKT, mTOR, p-PI3K, p-AKT, p-mTOR, OGT, and O-GlcNAcylation proteins were assessed in OSCC cells exposed to a medium with different glucose concentrations and OGT knockdown. *n* = three independent experiments. Data were presented as the mean ± SD. *P* values were determined by one-way ANOVA. *P < .05, **P < .01, ***P < .001, ****P < .0001.

Given the contributions of the PI3K/AKT/mTOR pathway to proliferation, EMT, and apoptosis,^18^ we measured the expression levels of PI3K, p-PI3K, AKT, p-AKT, mTOR, and p-mTOR in SCC25 and CAL27 cells in high-glucose medium and standard glucose medium. Our results showed that high glucose concentrations increased p-PI3K, p-AKT, and p-mTOR (Figure 5A-C). In addition, the knockdown of OGT prevented the activation of the PI3K/AKT/mTOR pathway due to high glucose concentrations (Figure 5A-C). These results indicate that hyper-O-GlcNAcylation-mediated proliferation, metastasis, and apoptosis partially depend on the PI3K/AKT/mTOR pathways.

## Discussion

The prevalence of DM and cancer is increasing and has reached epidemic proportions globally.^19^, ^20^, ^21^ Thus, codiagnosis is not uncommon. In a national survey of patients in the United States, the prevalence of DM among newly diagnosed cancer patients was 8% to 18%.^22^ Thus, cancer in patients with DM is becoming a significant health issue^23^; however, research into the development of personalized treatments for these patients is limited.

In this study, we adopted a dual-methodological approach that integrates clinical retrospective analysis and in vitro cellular experiments to comprehensively investigate the impact of DM on OSCC progression and its underlying mechanisms. Firstly, through a clinical data analysis, we showed that DM is strongly associated with poor prognosis in patients with OSCC, and this aligns with the findings reported by Kao et al and Zhang et al.^24^^,^^25^ Many studies have been conducted on the mechanisms of the association between DM and malignant tumours. DM is a persistent hyperglycaemic condition.^26^ Glucose is transported within cancer cells in a high-glucose environment, resulting in the activation of a variety of intracellular signalling pathways that promote malignant tumour progression. In vitro experiments, we examined the malignant phenotype of the tumours and found that high glucose promoted the proliferation, invasion, and migration of OSCC cells and inhibited apoptosis, which is consistent with the results reported by Su et al.^27^ A clinical study revealed that the likelihood of distant metastasis and recurrence after surgery in patients with poor glycaemic control was significantly increased compared to those with good glycaemic control.^28^ Therefore, it would be clinically beneficial to maintain the glycaemic control of patients with OSCC and DM within an acceptable range in the perioperative period.

Increased glucose intake contributes to increased HBP flux. Thus, as a product of HBP flux, O-GlcNAcylation levels rise in response to elevated glucose. Previous studies have shown that O-GlcNAcylation promotes head and neck squamous carcinoma cell proliferation and cell migration and inhibits apoptosis.^29^ However, the role of O-GlcNAcylation in OSCC is controversial. One study showed that O-GlcNAcylation was significantly upregulated in OSCC tissues and correlated with tumour size.^30^ In contrast, another study showed that, unlike other types of cancer, O-GlcNAcylation levels were not significantly increased in OSCC tissues or OSCC cells and were not associated with the histologic grade of OSCC.^31^ In our study, O-GlcNAcylation and OGT expression levels were significantly elevated in the tissues of patients with OSCC and DM. In addition, high-glucose treatment induced the upregulation of O-GlcNAcylation levels in OSCC cells, consistent with the findings of Wongkham et al’s^32^ cholangiocarcinoma study. In addition, we found that OGT knockdown and the OGT inhibitor OSMI-1 attenuated the effect of high glucose to increase OSCC proliferation and metastasis and promoted apoptosis. These results support our hypothesis that a sustained high-glucose environment promotes the malignant progression of OSCC cells via O-GlcNAcylation and may be the first evidence that hyper-O-GlcNAcylation is associated with OSCC progression. These findings suggest that O-GlcNAcylation exhibited considerable potential and possible clinical validity as a target for OSCC.

The PI3K/AKT/mTOR signalling pathway is the most frequently activated pathway in human cancers.^33^ This signalling pathway promotes cell adhesion, growth, migration, invasion, angiogenesis, and antiapoptotic pathways by phosphorylating downstream target proteins.^34^ O-GlcNAcylation can crosstalk with the phosphorylation of adjacent sites. Hyper-O-GlcNAcylation stimulates the activation of PI3K/AKT/mTOR signalling by upregulating AKT Ser473 phosphorylation in a variety of cancer cancers, including colorectal, gastric, and thyroid anaplastic cancer.^35^, ^36^, ^37^ Ju et al^38^ reported that the knockdown of OGT in pre-B acute lymphocytic leukaemia downregulated the phosphorylation of AKT Ser473, slowed the proliferation of Nalm-6 cells, and induced their apoptosis. Consistently, we observed that high glucose significantly increased PI3K, AKT, and mTOR phosphorylation in OSCC cells, and potent OGT inhibition partially prevented high glucose-induced increases in PI3K/AKT/mTOR pathway activity.

Research is needed to develop personalized treatments for patients with OSCC and DM. In this patient population, a healthy lifestyle, including no current smoking, moderate alcohol consumption, and a healthy diet, is an effective way to improve cancer prognosis.^39^, ^40^, ^41^, ^42^, ^43^ Targeted therapies that block O-GlcNAcylation should also be considered. As a widely used antidiabetic drug, metformin inhibits the expression of O-GlcNAcylation and OGT, thereby suppressing cancer cell proliferation and promoting apoptosis.^44^^,^^45^ Thus, metformin may be a potent O-GlcNAcylation-inhibiting drug with potential anticancer effects. However, it is important to note that strategies targeting global O-GlcNAcylation should be cautiously implemented, as it is also essential for normal cellular physiology. Therefore, the development of O-GlcNAcylation inhibitors to specific targets is necessary to improve the efficacy of tumour therapy. In recent years, many novel nanomaterials have been used as drug carriers for treating tumours.^46^ These drug delivery systems could provide efficient delivery and the controlled release of O-GlcNAcylation inhibitors.^47^ RNA aptamers, nanobody-OGT/O-GlcNAcase tools, and competitive peptides make it possible to add or remove O-GlcNAcylation from specific modification sites in target proteins.^48^, ^49^, ^50^ The development of these strategies could be extended to targeting O-GlcNAcylation on various targets and provide opportunities to design anticancer therapies.

However, the study’s limitations should also be acknowledged. First, larger multicentre/multicountry studies should be conducted to include more demographically diverse populations and ensure the credibility of the study results. In addition, a binary categorization of smoking and drinking status was used, which is not fully informative. The absence of former smoker and former drinker categories is a limitation, as tobacco and alcohol can modify risk levels. Furthermore, we could not confirm whether the in vivo effects of high glucose and O-GlcNAcylation on OSCC progression were consistent with the in vitro effects. Thus, a further study of the role of high glucose and O-GlcNAcylation in experimental animals is needed. Finally, the O-GlcNAcylation substrate and modification sites were blurred in OSCC tissues from DM patients and need further investigation.

## Conclusion

In summary, our results demonstrate that DM status was associate with poor prognosis in patients with OSCC. We provide a novel mechanism to address the protumoural properties of the high-glucose microenvironment in OSCC. The results revealed the protumoural properties of high glucose in OSCC, which are mainly mediated by O-GlcNAcylation and responsive to high glucose, upregulated by high glucose, and activate the PI3K/AKT/mTOR pathway, thereby promoting malignant proliferation and metastasis and inhibiting apoptosis. Thus, targeting O-GlcNAcylation is expected to be successfully applied to treat OSCC and other types of cancer in patients with DM.

## Author contributions

Zhuang Zhu: Conceptualization; data curation; formal analysis; investigation; writing- original draft. Wenhao Ren: Investigation; methodology; funding acquisition. Xiaohan Yan: Formal analysis; visualization; writing- review and editing. Shaoming Li: Investigation; methodology; resources. Jingjing Zheng: Methodology; visualization; funding acquisition. Keqian Zhi: Funding acquisition; supervision. Ling Gao: Supervision; project administration; writing – review and editing; funding acquisition.

## Ethics statement and consent to participate

The experimental protocols were approved by the Ethics Committee of the Affiliated Hospital of Qingdao University. Informed consent was obtained for studies involving human OSCC tissues.

## Funding

This study was funded by the National Natural Science Foundation of China (No. 42176097 [K.Q.Z.]), the Natural Science Foundation of Shandong Province (No. ZR2021MD065 [K.Q.Z.], ZR2021MH305 [J.J.Z.], ZR2022MH223 [W.H.R.]), Taishan Scholars Foundation of Shandong Province (No. tsqn202306397 [L.G.]), State Administration of Traditional Chinese Medicine Science and Technology Department Concentration of Science and Technology Project (GZY-KJS-SD-2023-078 [K.Q.Z.]), Shandong Province Medical Health Science and Technology Project (202308021296[K.Q.Z.]).

## Conflict of interest

The authors state that they have no potential conflicts of interest.

## Appendix Supplementary materials

**Fig. S1** (A and B) Quantification of the data in Figure 2J. (C) Quantification of the data in Figure 2I. (D) Quantification of the data in Figure 4C. (E and F) Quantification of the data in Figure 4I. (G) Quantification of the data in Figure 4H. *n* = three independent experiments. Data were presented as the mean ± SD. *P* values were determined by two-sided *t* tests (A, B, C, G) or one-way ANOVA (D, E, F). **P* < .05, ***P* < .01, ****P* < .001. Image, image 1

**Fig. S2** OSMI-1 inhibits proliferation and metastasis and enhances apoptosis in OSCC cells. The proliferation of SCC25 and CAL27 cells treated with or without 20 μM OSMI-1 was analysed using the (A and B) CCK-8 assay. (C and D) Wound healing and (E and F) transwell assays were used to detect the migratory and invasive abilities of OSCC cells after treatment with 50 μM OSMI-1 in 25 mM glucose. Scale bars: 200 μm. Expression of OGT and representative EMT and cell apoptosis markers of OSCC cells treated with or without 20 μM OSMI-1 for 24 hours were examined using (G-I) Western blot analysis. *n* = three independent experiments. Data were presented as the mean ± SD. *P* values were determined by two-way ANOVA (A and B) or the two-sided *t* test (C, D, F, H, I). **P* < .05, ***P* < .01, ****P* < .001, *****P* < .0001. Image, image 2

## Footnotes

Supplementary material associated with this article can be found in the online version at doi:10.1016/j.identj.2025.103897.
