# Supplementary figures and images for: High-Glucose Microenvironment Accelerates Malignant Progression Via O-GlcNAcylation in Oral Squamous Cell Carcinoma
Source: Int Dent J. 2025 Oct 8;75(6):103897. doi: 10.1016/j.identj.2025.103897 (PMC12538696; doi:10.1016/j.identj.2025.103897)

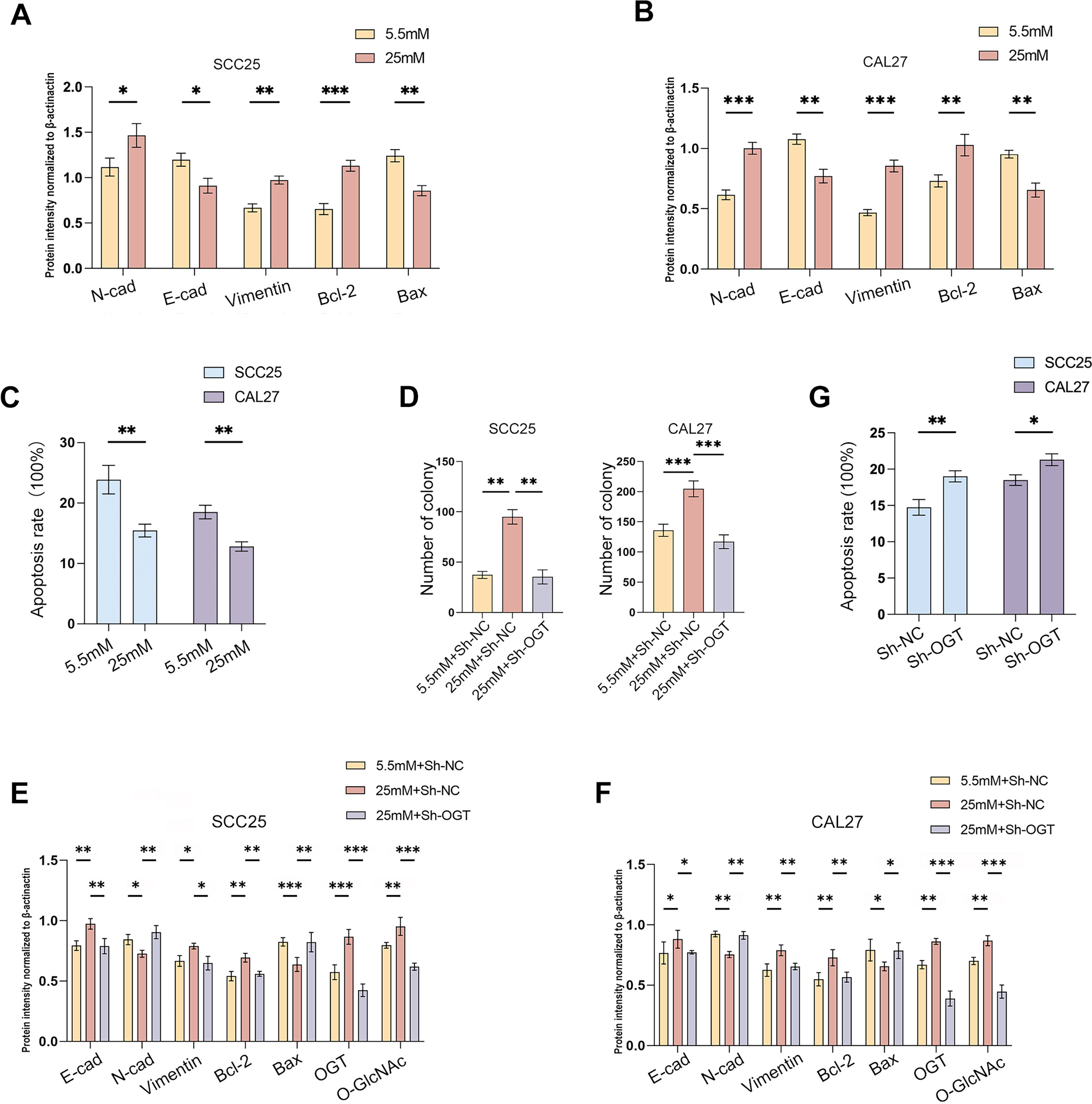

Supplement: Supplementary file 1 — Fig. S1 (A and B) Quantification of the data in Figure 2J. (C) Quantification of the data in Figure 2I. (D) Quantification of the data in Figure 4C. (E and F) Quantification of the data in Figure 4I. (G) Quantification of the data in Figure 4H. n = three independent experiments. Data were presented as the mean ± SD. P values were determined by two-sided t tests (A, B, C, G) or one-way ANOVA (D, E, F). *P < .05, **P < .01, ***P < .001. [file mmc1.jpg]

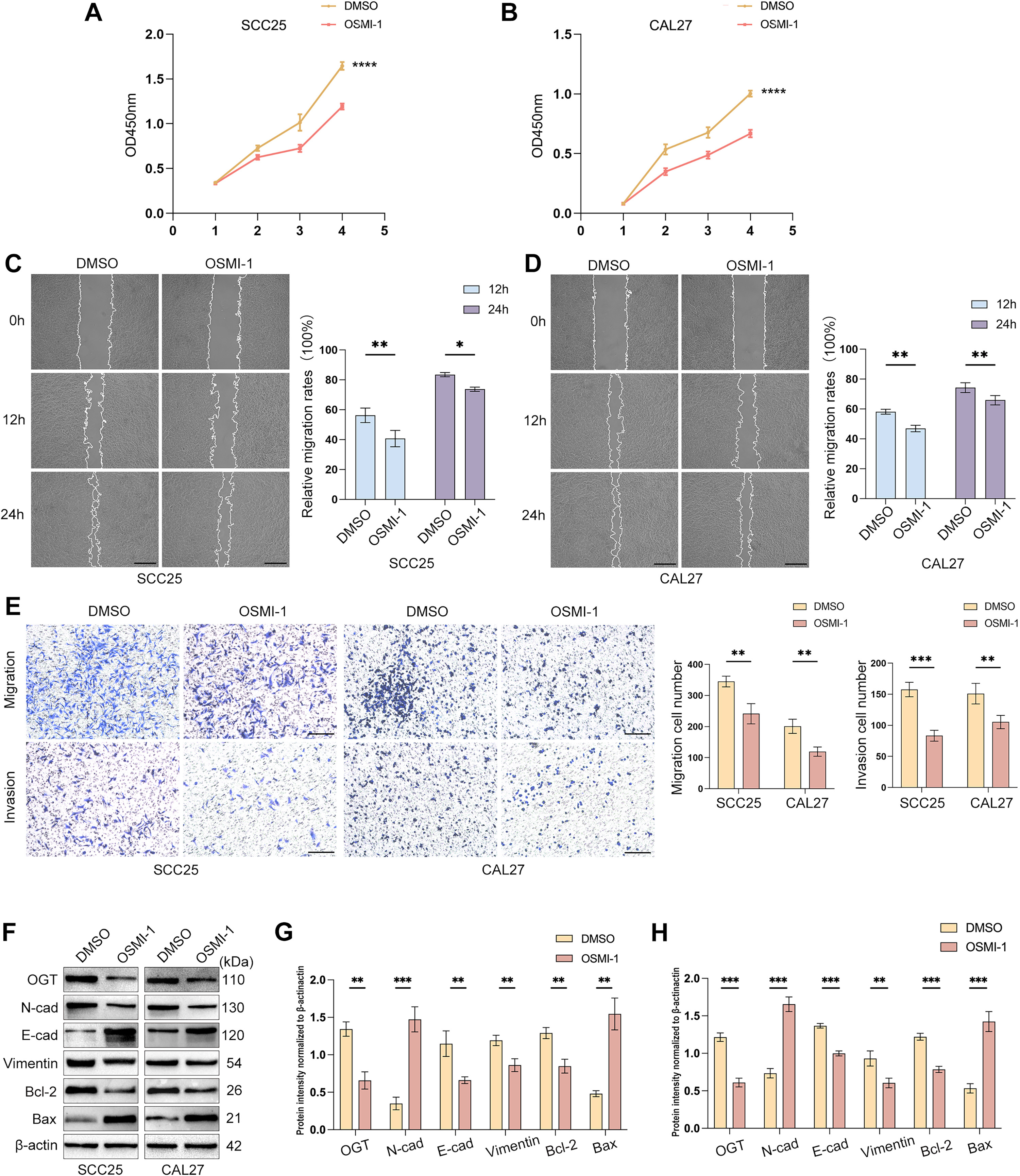

Supplement: Supplementary file 2 — Fig. S2 OSMI-1 inhibits proliferation and metastasis and enhances apoptosis in OSCC cells. The proliferation of SCC25 and CAL27 cells treated with or without 20 μM OSMI-1 was analysed using the (A and B) CCK-8 assay. (C and D) Wound healing and (E and F) transwell assays were used to detect the migratory and invasive abilities of OSCC cells after treatment with 50 μM OSMI-1 in 25 mM glucose. Scale bars: 200 μm. Expression of OGT and representative EMT and cell apoptosis markers of OSCC cells treated with or without 20 μM OSMI-1 for 24 hours were examined using (G-I) Western blot analysis. n = three independent experiments. Data were presented as the mean ± SD. P values were determined by two-way ANOVA (A and B) or the two-sided t test (C, D, F, H, I). *P < .05, **P < .01, ***P < .001, ****P < .0001. [file mmc2.jpg]
